# Teaching Pharmacovigilance to Healthcare Students: Identifying Gaps and Opportunities for Improvement

**DOI:** 10.3390/pharmacy9030147

**Published:** 2021-08-26

**Authors:** Ana Seselja Perisin, Josipa Bukic, Doris Rusic, Dario Leskur, Josko Bozic, Ante Mihanovic, Marino Vilovic, Tin Cohadzic, Darko Modun

**Affiliations:** 1 Department of Pharmacy, University of Split School of Medicine, Soltanska 2, 21 000 Split, Croatia; aperisin@mefst.hz (A.S.P.); jbukic@mefst.hr (J.B.); drusic@mefst.hr (D.R.); dleskur@mefst.hr (D.L.); ljekarnasdz@ljekarnasdz.hr (A.M.); tincohi@gmail.com (T.C.); 2 Department of Pathophysiology, University of Split School of Medicine, Soltanska 2, 21 000 Split, Croatia; josko.bozic@mefst.hr (J.B.); mvilovic@mefst.hr (M.V.); 3 Split-Dalmatia County Pharmacy, Kneza Ljudevita Posavskog 12 b, 21 000 Split, Croatia

**Keywords:** pharmacovigilance, healthcare students, survey, knowledge

## Abstract

The literature indicates that the limited pharmacovigilance knowledge demonstrated by healthcare professionals is the main reason for the underreporting of adverse drug reactions. Therefore, the main objective of this study was to investigate pharmacy, dental and medical students’ knowledge and attitudes to pharmacovigilance and pharmacovigilance education. The cross-sectional questionnaire study was conducted at the University of Split School of Medicine in November 2020. In total, 350 students participated in the study. The results have shown that pharmacy students showed a significantly higher knowledge score compared to dental and medical students (*P* < 0.001). In total 92.2% of pharmacy, 21.8% of dental and 70.8% of medical students had knowledge of patients’ involvement in adverse drug reactions, reporting (*P* < 0.001). Interestingly, only 44.3% of all students knew that adverse drug reactions could be reported using a mobile application. Moreover, significantly more pharmacy students (74.4%) were aware of the adverse drug reactions monitoring center in Croatia, with 47.5% of dental and 39.2% of medical students correctly identifying it (*P* < 0.001). The results showed that most students felt that pharmacovigilance was not adequately covered in curricula; therefore, there is a great need to increase the knowledge and awareness of pharmacovigilance among students aspiring to become future healthcare professionals, and improve their reporting practice in clinical future.

## 1. Introduction

According to the World Health Organization (WHO), pharmacovigilance is defined as the science and activities related to the detection, assessment, understanding and prevention of adverse drug reactions or other drug-related problems. Moreover, pharmacovigilance aims to enhance patient safety and support public health programs by providing reliable information for the efficient assessment of the benefit-risk profile of drugs and vaccines. Furthermore, as the reporting of adverse drug reactions is one of the indicators of healthcare quality, healthcare professionals should report adverse drug reactions as part of their everyday clinical practice [1,2] 

Pharmacovigilance education is also offered to healthcare professionals as part of their continuous education. Previous studies showed that pharmacovigilant educational interventions, such as workshops, significantly improved the knowledge scores and attitudes of healthcare professionals. Moreover, educational programs with continuous medical education credits were recognized as a factor which facilitated the reporting of adverse drug reaction [3,4,5,6]. However, another problem is the transition from education on pharmacovigilance to practice. Research showed this transition was still not adequate and that the reporting practice could be further increased by improving access to reporting forms or by using user-friendly methods, such as electronic reporting and educational interventions, which should especially target the junior healthcare professionals. Moreover, healthcare professionals are less likely to report adverse drug reactions if they are well-known or if they are not categorized as serious. Therefore, education is needed to raise awareness and knowledge of adverse drug reaction reporting [7,8].

In order to ensure the appropriate pharmacovigilant practice of healthcare professionals, there is a need for education of both healthcare professionals and students. However, the introduction of pharmacovigilant education in university study programs in the field of biomedicine is in its formative stages in most countries of the world [9,10,11]. Based on previous research, it seems reasonable that the education of future health professionals at the level of study programs should be focused on three key aspects of pharmacovigilance: awareness, knowledge and reporting [10]. A review article by Reumerman et al., on student education in the field of pharmacovigilance, showed that students’ intentions to report adverse drug reactions and their attitudes about reporting suspected adverse drug reactions were positive, although most students said they felt insufficiently prepared to report the suspected adverse drug reactions themselves. Moreover, even medical students who had experience of clinical rotations, did not report adverse drug reactions themselves. In order to raise awareness of the importance of reporting suspected adverse drug reactions in the student population, the authors stated the need to introduce innovative pharmacovigilance education at university level [11].

Despite numerous educational activities and other initiatives aimed at increasing the adverse drug reaction reporting practice, the underreporting of healthcare professionals remains an issue. However, the majority of studies concluded that the limited pharmacovigilance knowledge of healthcare professionals was the main reason for underreporting [12,13,14,15,16,17,18]. Therefore, the main objective of this study was to investigate students’ knowledge and attitudes to pharmacovigilance and adverse drug reaction reporting.

## 2. Materials and Methods

### 2.1. Participants

In order to examine the pharmacovigilance knowledge and attitudes of students, a cross-sectional survey was conducted using an anonymous survey questionnaire. The study was conducted from 1 November to 30 November 2020. The processed data were collected online via Google Forms, the link of which was forwarded to all students of pharmacy, dental medicine and medicine departments at University of Split School of Medicine, with the help of student representatives of each study year. The study was approved by the Ethics Committee of University of Split School of Medicine (approval number: 2181-198-03-04-20-0001, approved date: 30 January 2020). Participation was completely voluntary and respondents were informed that by completing the questionnaire they gave their informed consent to participate in this study. Students received no compensation for participation in the study. The questionnaire used in this research was adopted from the questionnaire published by Alkayyal et al. [19]. The original questionnaire was first translated into Croatian and then translated back into English by a native English speaker. Pilot study conducted among 10 students of each study program ensured clarity of the questionnaire and suitability to the student population. Pilot study revealed that none of the questions were unclear to participants. Furthermore, it enabled evaluation of the time needed to fill out the questionnaire.

Sample size was calculated using differences in frequencies of correct answers in knowledge test about pharmacovigilance, between pharmacy and medical students from study by Alwhaibi et al., with alpha adjusted for multiple comparisons and set as 0.017 and power as 0.8 [20]. Necessary sample size was determined to be 342.

### 2.2. Questionnaire

The survey questionnaire consisted of four parts and contained a total of 34 items. Pilot study revealed that it took 10–15 min to complete the whole questionnaire. The first part of the questionnaire, comprised of 10 items, included the sociodemographic data such as gender, study program, year of study, grade point average, family member as healthcare professional, experience with drug use and adverse drug reactions. The second part of the questionnaire, which included 10 items, was composed of questions testing the knowledge of pharmacovigilance and the reporting of adverse drug reactions. Students were offered to answer with yes/no/I do not know. We have added two questions in this section of the questionnaire, in comparison to the original survey developed by Alkayyal et al. Students were asked whether patients were allowed to report adverse drug reactions and whether adverse drug reactions could be reported using mobile application. These questions were added as patients reporting and mobile applications enabled an increase in number of adverse drug reactions reports and students should have been familiar with this. Knowledge assessment included knowledge of the definitions of pharmacovigilance and adverse drug reactions, information on the national regulatory center for monitoring of adverse reactions, types of adverse drug reactions, familiarity with the form for reporting suspected adverse drug reactions and which adverse reactions should be reported. Students were given one point for each correct answer which made the maximum possible knowledge score 7 points.

The third part of the questionnaire, comprised of 8 items, determined students’ attitudes about the importance of reporting known adverse drug reactions, the importance of pharmacists, medical doctors or dentists in reporting, the obligation to report adverse drug reactions for all health professionals, and the motivation to report in future clinical practice. We added items regarding whether pharmacists were the most important professionals, as they reported the adverse drug reactions of over-the-counter (OTC) drugs. The fourth part consisted of 6 items and examined the coverage of the topic of pharmacovigilance by the curriculum and the readiness of students to report suspected adverse drug reactions. For instance, students were asked whether they believed that the topic of pharmacovigilance is not well-covered in their school curriculum or if pharmacovigilance should be included as a core topic in their education. In the third and fourth parts of the questionnaire, statements were rated on a Likert scale (1 = strongly disagree, 2 = disagree, 3 = neither disagree nor agree, 4 = agree, 5 = strongly agree).

### 2.3. Statistical Analysis

MedCalc (version 19.2.6, MedCalc Software, Ostend, Belgium) statistical program was used for data analysis. The results of the study were presented as whole numbers and percentages and as a median and interquartile range (IQR), where appropriate. A chi-square test was used to compare the demographic characteristics of students, and Kruskal–Wallis with post hoc Dunn test was used to compare the level of knowledge and attitudes about pharmacovigilance. The level of statistical significance was set at *P <* 0.05.

## 3. Results

In total, 350 students participated in the present study. More than one third of participants were pharmacy students (*N* = 129, 36.9%), followed by dental (*N* = 101, 28.9%) and medical students (*N* = 120, 34.3%). Pharmacy students reported adverse drug reaction in the greatest proportion (17.1%), while only three (3%) dental students reported adverse drug reations by the time the study was conducted. All students, regardless of study program, reported a similar frequency of medication use and experienced drug reaction (*P* = 0.540, *P* = 0.928, respectively). The characteristics of the study participants are presented in Table 1.

**Figure 1:**
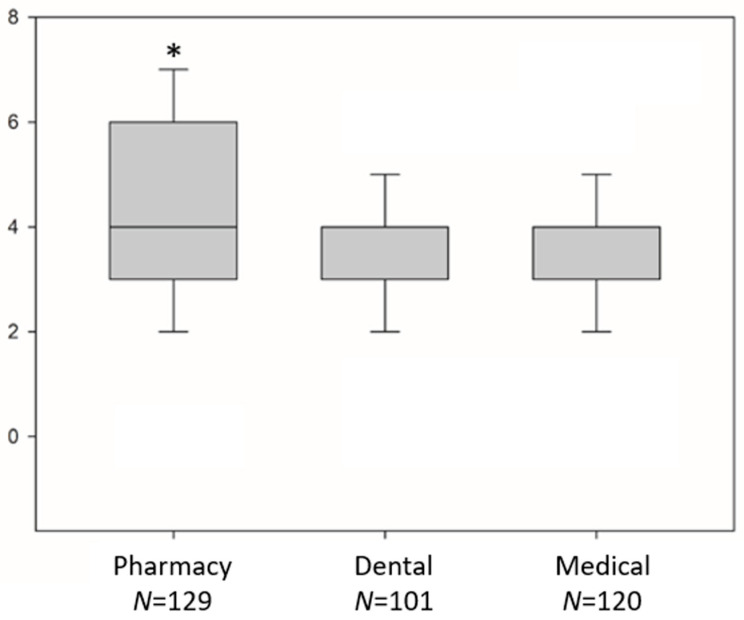
Students’ knowledge scores across study programs. Data are presented as median and interquartile range; knowledge score range (0–7). * Kruskal–Wallis test, with post hoc Dunn analysis, *P <* 0.05 Pharmacy vs. Dental, Pharmacy vs. Medical.

Pharmacy students showed a significantly higher knowledge score, a median value of 4 (3–6), compared to a dental value of 3 (3–4), and a medical value of 3 (3–4) (*P* < 0.001). (Figure 1). Moreover, only 44.3% of students knew that adverse drug reactions could be reported using a mobile application. Furthermore, 64.6% of all students knew that patients could report adverse drug reactions themselves, the majority of which were pharmacy students.

The majority of pharmacy students, 78.3%, compared to 62.4% dental and 58.3% medical students, knew about the different types of adverse drug reactions, *P* = 0.002. Moreover, significantly more pharmacy students (74.4%) were aware of the adverse drug reactions monitoring center in Croatia, with 47.5% of dental and 39.2% of medical students correctly identifying it (*P* < 0.001). Only 33.7% of all students had seen an adverse drug reaction reporting form. Almost half of pharmacy students (47.3%), compared to 27.7% of dental and 24.2% of medical students, had seen the adverse drug report form (*P* < 0.001).

Students’ attitudes on pharmacovigilance are presented in Table 2. There was no difference among students when agreeing that physicians were the most important healthcare professionals for reporting adverse drug reactions for prescription drugs (*P* = 0.482). All students were more likely to agree that pharmacists were the most important healthcare professionals to report adverse drug reactions of OTC drugs than prescription drugs. Moreover, pharmacy students were significantly more willing to report any adverse drug reaction in their future practice, compared to dental and medical students, (*P* < 0.001) and more frequently thought that adverse drug reaction reporting should be made compulsory for healthcare professionals (*P* = 0.002). Furthermore, pharmacy students were more familiar with the reporting obligations in clinical trials compared to other students.

## 4. Discussion

The results of this study indicate a need for greater teaching on topic of pharmacovigilance for healthcare students. Analysis shows that students are not confident in reporting adverse drug reactions and are not sure how to report adverse drug reaction to the relevant authorities. There is an obvious need for practical teaching on pharmacovigilance as only 33.7% of all students have seen an adverse drug reactions report form. Based on this result it seems reasonable to implement a pharmacovigilance course in the curricula of their studies. This course should introduce students to basic knowledge of adverse drug reactions and the role of the adverse drug reactions monitoring centre in Croatia. Moreover, each student should be able to report reactions using both the standard report and a mobile application, after completing the course. It is also important that students learn that patients can report their own adverse drug reactions, so that, in their future clinical practice, they could encourage patients to report adverse drug reactions themselves.

This study demonstrates that pharmacy students are aware of their future role as main healthcare professionals for the pharmacovigilance of OTC drugs, as well as the fact that they are the most familiar with the reporting obligations for clinical trials. Furthermore, a higher proportion of pharmacy students compared to other included students know how to report adverse drug reaction and have reported adverse drug reaction themselves. However, compared to previous studies, where 68.5% of pharmacy students stated that they knew how to report adverse drug reactions, there is a need for an increase in the reporting knowledge for all students at the University of Split School of Medicine [21]. Moreover, pharmacy students showed a higher knowledge score compared with dental and medical students. This is similar to the results of the study conducted by Bepari et al. where pharmacy students showed a better mean knowledge score and practice skills, compared to medical and nursing students [22]. Furthermore, pharmacy students showed modest confidence in their preparedness to report adverse drug reactions. Unfortunately, this pattern is seen among other pharmacy students. In a study conducted among Korean pharmacy students, only 30.7% of students felt they had gained sufficient knowledge on adverse drug reaction reporting for future practice [23].

In this study 74.4% of pharmacy students were aware of the adverse drug reactions monitoring center in Croatia, compared to only 59.1% Saudi pharmacy students, which is an encouraging finding [19]. However, a significantly lower awareness was observed for dental and medical students.

The observed gap in students’ knowledge of pharmacovigilance is likely due to differences in pharmacy, dental and medical curricula, but should be addressed as the topic of pharmacovigilance is important for all healthcare professionals. A recent systematic review confirms that healthcare students differ in their knowledge and attitudes towards adverse drug reaction reporting and pharmacovigilance [20]. Therefore, it seems reasonable to improve courses that offer pharmacovigilance education, or to increase their duration. Other than by changes in curriculum of study programs, students can be offered pharmacovigilance education outside of college. For instance, Uppsala Monitoring Centre developed a micro learning-based module offered as e-learning. The use of e-learning could increase the availability of pharmacovigilance education and can be offered as part of a continuous medical education [24]. Furthermore, interdisciplinary education, workshops and courses may be offered to students on topics valuable to different healthcare professionals [25]. The importance of education on pharmacovigilance was recognized at Pharmacy studies at the University of Split School of Medicine. Namely, during the fifth year of study, as part of their professional training in the Community Pharmacies of the Split-Dalmatia County, students are required to report one adverse drug reaction with their mentor via a paper form and one online. Additionally, after professional training, students take an objectively structured clinical exam, which contains the station ‘Safe use of drugs’. At the mentioned station, students have the task of contacting a physician if necessary (if it is a serious adverse drug reaction), and to notice, prevent and react to medication errors, and to identify and report adverse drug reactions of medications to the competent authority [26].

Our results showed that 65% of all students had knowledge that patients could report adverse drug reactions. Moreover, in pharmacy students this proportion was above 90% which suggests that pharmacy students were more knowledgeable about patient reporting, compared to their dental and medical colleagues. This calls for not only greater education in pharmacovigilance among healthcare students, but also for greater campaigns toward the general public on monitoring adverse drug reactions. It should be noted that patients do not have the opportunity to report adverse drug reactions in numerous countries worldwide, although this is possible in Croatia. Moreover, in a study by Matos et al., patients were not allowed to report adverse drug reactions in 34 out of 141 countries which participated in the study. However, research showed that patients were motivated to report adverse drug reactions, particularly those that affected their quality of life. Furthermore, patient reporting brings a new depth of information for adverse drug reactions, mainly regarding symptoms description and psychobiosocial impact, and also promotes the involvement of patients in their pharmacotherapy [27,28,29,30,31].

Moreover, a small proportion of students were familiar with the reporting of adverse drug reactions using a mobile application. It is important to ensure that pharmacovigilance education offers students with the latest knowledge on reporting adverse drug reactions, e.g., via a mobile application, as this method was previously described as user-friendly and helps to augment pharmacovigilance activities among patients. Yet, the use of the application to date seems modest in comparison to other reporting methods. However, it seems reasonable to expect that this reporting method will grow in importance as a younger generation of patients matures [32].

All students recognized pharmacists as most important healthcare professionals to report adverse drug reactions for OTC drugs. Research shows that pharmacists indeed are most prominent reporters of adverse drug reactions for OTC drugs. However, all healthcare professionals should be aware that OTC drugs could also cause adverse drug reactions [33]. Students identified physicians as most important healthcare professionals for monitoring adverse drug reactions for prescription drugs; however, as pharmacists are the most accessible healthcare workers [34], their role in pharmacovigilance of chronic treatments should not be overlooked.

Our study has several limitations. First of all, it is a single-center study as it was conducted only at the University of Split School of Medicine. Furthermore, another limitation is the use of the electronic survey. Students were asked not to seek references and participation was voluntary and anonymous, so we believe that they followed the provided instructions. Due to the COVID-19 pandemic this was the only option for distributing the surveys. Yet, even with all the limitations, the results of this study adds to the body of research requesting the increased attention toward pharmacovigilance

## 5. Conclusions

The results showed that most students feel that pharmacovigilance is not adequately covered in curricula; therefore, there is a great need to increase the knowledge and awareness of pharmacovigilance in students as future healthcare professionals, and improve their reporting practice in the clinical future. It would be interesting to investigate how students’ attitudes and knowledge of pharmacovigilance translates into future practice. Future studies should involve educational interventions which would meet the needs of future healthcare professionals. This study confirms previous research that pharmacy students have the greatest knowledge of pharmacovigilance and that there is a need for the integration of education on this topic among healthcare programs. This is further stressed in times of the rapid development of treatment options, such as vaccines for COVID-19, as novel therapies require great vigilance in practice.

## Figures and Tables

**Table 1:** Students’ characteristics.

Participants	Pharmacy Students *N* (%)	Dental Students *N* (%)	Medical Students *N* (%)	Total *N* (%)	*P* Value *
Number of students	129 (36.9)	101 (28.9)	120 (34.3)	350 (100)	
Female gender	112 (86.8)	79 (78.2)	81 (67.5)	272 (77.7)	0.001
Family members who are healthcare professionals	40 (31.0)	38 (37.6)	45 (37.6)	123 (35.1)	0.465
Use of medication (if ever)	115 (89.1)	93 (92.1)	105 (87.5)	313 (89.4)	0.540
Adverse drug reaction experienced during use	43 (33.3)	36 (35.6)	42 (35.0)	121 (34.6)	0.928
Adverse drug reaction reported	22 (17.1)	3 (3.0)	14 (11.7)	39 (11.1)	0.003
Knowledge of mobile application reporting route	85 (65.9)	9 (8.9)	61 (50.8)	155 (44.3)	0.001
Knowledge of patients reporting	119 (92.2)	22 (21.8)	85 (70.8)	226 (64.6)	0.001

* chi square test; data are presented as numbers (proportion).

**Table 2:** Students’ attitudes of pharmacovigilance.

Attitude Item	Pharmacy (*N* = 129)	Dental (*N* = 101)	Medical (*N* = 120)	*P* Value *
Reporting of known adverse drug reactions makes no significant contribution to the reporting system.	2 (1–3)	2 (2–3)	2 (2–3)	0.192
I believe a pharmacist is one of the most important healthcare professionals to report the adverse drug reactions of OTC drugs.	5 (4–5) ^ab^	4 (3–4)	4 (3–4)	<0.001
I believe a pharmacist is one of the most important healthcare professionals to report the adverse drug reactions of prescription drugs.	4 (4–5) ^ab^	3 (3–4) ^b^	3 (2–4) ^a^	<0.001
I believe a physician is one of the most important healthcare professionals to report the adverse drug reactions of prescription drugs.	4 (4–4)	4 (3–4)	4 (3.5–4)	0.482
I believe a dentist is one of the most important healthcare professionals to report the adverse drug reactions of prescription drugs.	4 (3–4)	4 (2.75–4)	4 (3–4)	0.631
I believe serious and unexpected reactions that are not fatal or life-threatening during clinical trials must not be reported.	1 (1–2) ^ab^	2 (1–3)	2 (1–4)	<0.001
I’m willing to report any adverse drug reaction in my future practice.	5 (4–5) ^ab^	4 (4–5) ^b^	4 (4–5) ^a^	<0.001
Adverse drug reaction reporting should be made compulsory for healthcare professionals.	5 (4–5) ^ab^	4 (4–5)	4 (4–5)	0.002

* Kruskal–Wallis test, with post hoc Dunn analysis, ^a^ significant *P* < 0.05 vs. dental, ^b^ significant *P* < 0.05 vs. dedical; data are presented as median (interquartile range), Likert scale from 1-strongly disagree to 5-strongly agree; OTC: over-the-counter. Students’ attitudes of pharmacovigilance education and coverage of the subject in their curriculum are presented in Table 3. Interestingly, medical students most frequently stated they did not believe that the topic of pharmacovigilance was well-covered in the school curriculum but were also least likely to agree that it should be taught to senior students. Pharmacy students had the greatest confidence for reporting in their future practice (*P* < 0.001). Dental students in the greatest proportion agreed that they have no idea how to report adverse drug reactions to the relevant authorities (median = 4, IQR 3-4; *P* = 0.001) (Table 3).

**Table 3:** Students’ attitudes of pharmacovigilance education.

Attitude Item	Pharmacy (*N* = 129)	Dental (*N* = 101)	Medical (*N* = 120)	*P* Value *
I believe that the topic of pharmacovigilance is not well-covered in my school curriculum.	3 (3–3.25) ^ab^	3 (3–4) ^b^	4 (3–4) ^a^	<0.001
Pharmacovigilance should be included as a core topic in formal education.	4 (4-5) ^ab^	4 (3–4)	4 (3–4)	<0.001
Students can perform adverse drug reactions reporting during their clerkship.	4 (4-5) ^ab^	4 (3–4)	4 (3–4)	<0.001
Information on how to report adverse drug reactions should be taught to senior students.	5 (4-5) ^ab^	4 (4–4)	4 (3–4)	<0.001
With my present knowledge, I am very well prepared to report any adverse drug reactions in my future practice.	3 (2–4) ^b^	2 (2–3) ^b^	3 (2–4) ^a^	<0.001
I do not have any idea of how to report adverse drug reactions to the relevant authorities.	2 (1–4) ^a^	4 (3–4) ^b^	3 (2–4) ^a^	0.001

* Kruskal–Wallis test, with post hoc Dunn analysis, ^a^ significant *P* < 0.05 vs. dental, ^b^ significant *P* < 0.05 vs. medical; data are presented as median (interquartile range), Likert scale from 1-strongly disagree to 5-strongly agree.

## Data Availability

The datasets generated during and/or analyzed during the current study are available from the corresponding author on reasonable request.
